# FlaxPack: Tailored Natural Fiber Reinforced (NFRP) Compliant Folding Corrugation for Reversibly Deployable Bending-Active Curved Structures

**DOI:** 10.3390/polym16040515

**Published:** 2024-02-14

**Authors:** Kevin Saslawsky, Christian Steixner, Michael Tucker, Vanessa Costalonga, Hanaa Dahy

**Affiliations:** 1 ITECH Master’s Program, University of Stuttgart, Keplerstr. 11, 70174 Stuttgart, Germany; ksas123@gmail.com (K.S.); cgsteixner@gmail.com (C.S.); mpt3cd@virginia.edu (M.T.); 2 BioMat@Stuttgart (Bio-Based Materials and Materials Cycles in Architecture), Institute of Building Structures and Structural Design, University of Stuttgart, Keplerstr. 11, 70174 Stuttgart, Germany; contact@hanaadahy.com; 3 BioMat@Copenhagen (Bio-Based Materials and Materials Cycles in the Building Industry Research Centre), Institute of Planning, TECH Faculty Technical Faculty for IT & Design, Aalborg University, Denmark AC Meyersvænge 15, 2450 Copenhagen, Denmark; 4 Faculty of Engineering, Department of Architecture (FEDA), Ain Shams University, Cairo 11517, Egypt

**Keywords:** Natural Fiber Reinforced Polymers (NFRP), tailored fibre placement (TFP), moldless fabrication, flat pack, bending active structures, lightweight structures

## Abstract

As the use of Natural Fiber Reinforced Polymers (NFRPs) become increasingly popular in the built environment, steps in established workflows, including molding and transportation, continue to impose constraints on what is possible in the material’s fabrication process. This research builds on previous studies of moldless fiber composites using tailored fiber placement (TFP) as a fabrication method. By integrating compliant folding mechanisms into the flat preform to give shape to the final desired geometry this research replaces all dependencies on molds and formworks during the resin curing process with programmed formal deformations. The desired geometry is digitally simulated from its two-dimensional state into its resultant three-dimensional state and then subsequently structurally analyzed. The flat pack components are material efficient and can be transported flat to the site for their final assembly into their programmed geometry. This form is locked into its bent active state through the use of a simple drawstring that can later be removed to revert the form back into its flat state. This method is demonstrated through the digital fabrication of a stool where flat-packed elements can be deployed into elegant solutions that embody structure, material, and form simultaneously.

## 1. Introduction

The environmental impact of the construction industry is staggering, with commercial construction alone accounting for over 35% of global energy consumption and utilizing nearly 45% of global resources. Notably, cement, contributing 8% to annual global carbon dioxide production, emerges as a significant environmental concern [1,2,3]. This emphasizes the critical need for sustainable practices and innovations within the construction sector to mitigate its adverse environmental effects.

**Figure 1:**
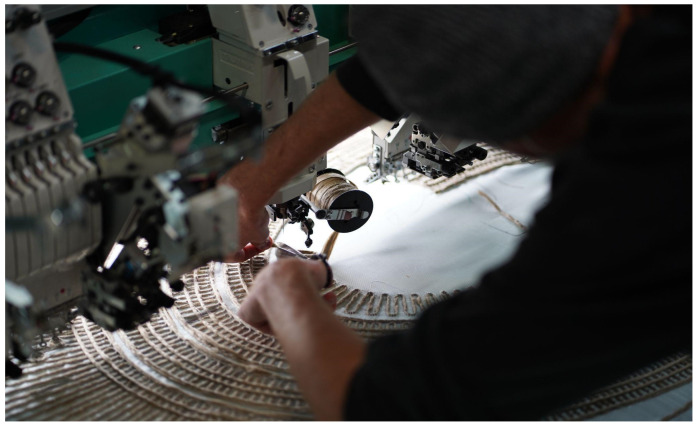
Photograph of tailored fiber placement fabrication production using a Tajima dual headed embroidering machine.

Tailored Fiber Placement (TFP), an automated textile fabrication method originating from the aerospace industry, offers a promising solution (Figure 1). As an additive manufacturing method, TFP strategically places reinforcement material where structurally necessary, resulting in lightweight structures with a high strength-to-weight ratio [4,5]. This, in turn, facilitates reduced material usage for structures and the subsidiary elements that often support them. TFP techniques employ digital design-to-production workflows for precision, efficiency, and mass customization [6].

**Figure 2:**
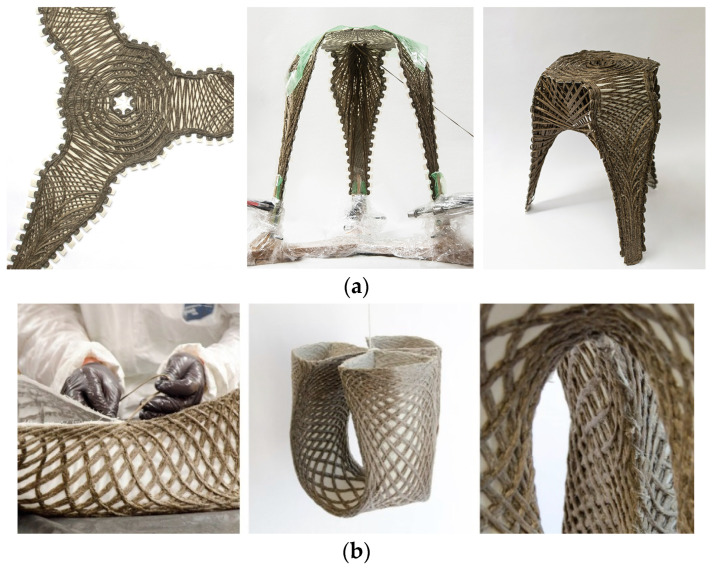
Previous State of the Art projects presented different strategies to eliminate single-use molds or frames in the TFP fabrication process. The FlaxFlex stool (**a**) used Classical Lamination Theory to produce a flat preform that could be bent to the desired angle after curing. The winded fibers served as locking mechanism for the bent active preform. The FibrFoldr stool (**b**) used a hanging method to cure the stool in the desired shape.

The potential for transferring this technology to architecture has already been demonstrated [6,7,8]. Notably, the projects FlexFlax [6] and FibrFoldr [7] propose strategies to eliminate wasteful single-use molds or frames in the forming process (Figure 2). On a larger scale, these contributions would exponentially lower costs and yield material savings for the construction of architectural use cases. The creators of the FlexFlax stool devised a strategy to minimize formwork by creating a preform that could be manipulated after curing. Applying principles of Classical Lamination Theory, the project tailored the fibers to produce stiff and bending zones, allowing the preform to remain malleable even after curing. The cured preform was then bent at the desired angle, and a syntax of continuous fibers was wound to lock the bent active structure in place [6]. The FibrFoldr stool introduced an alternative method to minimize formwork while maintaining a high degree of customization. Complex spatial geometries were achieved by tailoring fibers into a substrate to program anisotropy and curve folding patterns into a flat reinforcement. After being infused with resin, the preform was curve folded and suspended atop bent scaffolding rods, allowing gravity to shape the material during curing [7].

**Figure 3:**
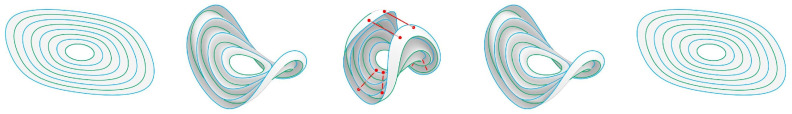
Research concept of a flat curve pattern that is folded and held together with drawstrings and subsequently released and unfolded into its original flat state.

This research advances these precedents by introducing zones of fiber-reinforced elastomers to curve folding composite structures [9]. While FlexFlax and FibrFoldr proposed effective strategies for moldless fabrication, this study aims to transcend their limitations by implementing the possibility for deployable and reversible assembly. Design for Disassembly (DfD), a pivotal concept in the construction industry, addresses concerns around resource consumption and low recycling rates. By definition, DfD is the design of buildings to facilitate future changes and dismantlement (in part or whole) for the recovery of systems, components, and materials [10]. This research approaches DfD through the lens of transportation logistics. Thanks to the ability to transport NFRP structures in their flat state, larger quantities of composite structures can be moved at a reduced volume offering cost savings and reduced transportation emissions. The reversibility offered by introducing elastomeric fold zones in curve folded NFRP perpetuates the ease of disassembly and efficient transportation towards a path for sustainable reuse (Figure 3).

Regarding design strategies, novel hinge-like curve folding methods are investigat-ed to allow for the assembly and disassembly of TFP preforms, which in turn will open new form-finding morphologies. This new found design space is explored through scaled prototyping (Section 2.4) accomplished by tailoring small sections of various folding patterns. Path planning (Section 2.5) is accomplished through rigorous parametric modeling and flexing of different patterns. Flat patterns are tested through particle and spring simulations (Section 2.6) and tested for viability via structural analyses while sim-ultaneously being vetted at a smaller scale via 3D printing (Section 2.7).

This approach offers a sustainable alternative to conventional systems by utilizing curve folded NFRP components in various architectural contexts. The workflow, which integrates computational design and simulation, curved fold forming, and the flexible and reversible application of materials, promises to deliver aesthetically elegant solutions embodying structure, material, and form simultaneously.

## 2. Materials and Methods

### 2.1. Tailored Fiber Placement (TFP)

For this research, a four stitch-headed TLMX-1202 TFP machine from TAJIMA was employed, situated at the BioMat department in the Institute of Built Structures and Structural Design (ITKE) at the University of Stuttgart. The machine features a maximum working area of 830 × 830 mm for each stitching head. The fundamental operation of TFP involves the ability of the stitching head to rotate freely by 360 degrees while the frame holding the base fabric moves in the x and y directions. To customize the fiber placement on the fabric, a stitch type parameter generates a final zig-zag pattern, effectively binding the fibers to the base material. Importantly, TFP machines are capable of producing intricate patterns within the tolerances of the material, offering a completely automated production process. A speed average of 500–600 stitches per minute was set. Polyester sewing yarn was used with Amann Serafil 120/2 for the upper and Amann Serafil 200/2 for the lower thread.

Non-twisted flax fiber rovings at a 2400 Tex linear density were selected for all production methods at the prototyping stage and sourced from the distributor Group Depestele (Teillage Vandecandelaere 5, rue de l’église, 14 540 Bourguebus, France). Flax Fibers were chosen due to their availability in Europe as well as for their high strength of 500–900 MPa, which exceeds that of many other natural fibers [7]. The fiber bundles were laid with a stitch width of 3.5 mm, resulting in a circular cross-section. The stitch length was constant at 7 mm. As a substrate, 106 g/m^2^ glass fiber textile from the distributor WELA (Tempelhofer weg 13, D-21502, Geesthacht, Germany) was chosen due to its availability and our previous experience with the material.

For impregnation, fully synthetic Epoxy Resins (3 parts EPIKOTE Resin MGS RIMR 235 + 1 part EPIKURE Curing Agent RIMH 237, Pot Life: 48 h, all produced by Hexion Inc. and provided by Hexion Stuttgart GmbH, Fritz-Müller-Straße 114, 73730 Esslingen am Neckar, Germany) were selected as the base matrix, due to their availability and previous experience with the material. A Pioneer basic vacuum table from the brand Columbus GmbH (Peter-Behrens-Platz 6 A-4020 Linz, Tabakfabrik, Germany), was used in the resin infusion process. The infused preforms were placed in plastic bags and vacuumed overnight while curing. To create the reversible hinge zones, a generic transparent silicone sourced from Bauhaus AG was applied in order to prevent resin from impregnating the substrate in specific locations.

### 2.2. Fabrication Constraints

**Figure 4:**
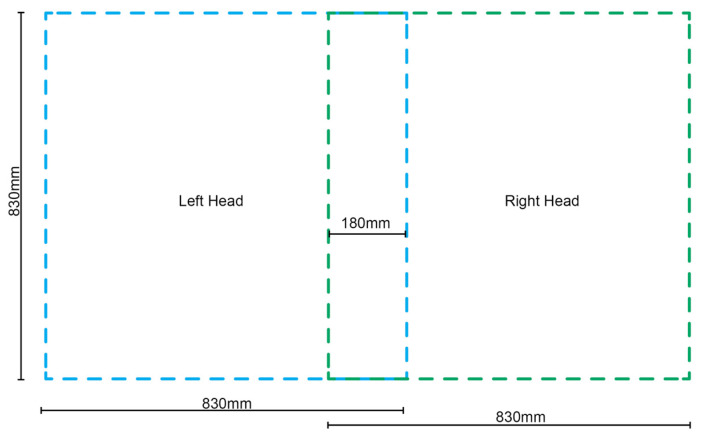
Diagram outlining the maximum tailored fiber placement work area of both tailoring heads along with the dimensionality of overlap between them.

One limitation of the TFP technique is its planar geometric outcome, necessitating methods to impart shape to the resultant preforms. The Tajima embroidering machine employed in this research features two tailoring and two embroidery heads mounted at equal intervals across its width. To optimize the build area, a single tailoring head was used at a time, avoiding duplicate components. During tailoring, the stitching heads remain stationary while the frame, holding the substrate, moves side to side or forwards and backwards. Each head can operate within an area of 830 mm by 830 mm, with a 180 mm overlap between them, as illustrated in Figure 4. Adherence to these size constraints is crucial when creating fiber paths to avoid exceeding the fabrication area.

Prior research, exemplified by FibrFoldr, proposed a method for producing patterns larger than a single tailoring head’s working area. This involved tailoring multiple sections separately and then realigning them for stitching within the machine’s working limits. In contrast, our approach utilizes both heads with distinct tailoring patterns on a single fabric piece, maximizing the machine’s build area. This involves establishing a global coordinate point in the lower left-hand corner of the physical substrate mounting frame, shared by two distinct files—one for the left region using the left head and one for the right region using the right head. The 180 mm-wide overlap zone between frame regions ensures continuous fibers. This method, leveraging both heads on different files with a shared global coordinate system, mitigates the risk of misaligning fiber paths between tailoring files. This overlapping technique is significant as the employed machine had only two tailoring heads, whereas most manufacturers offer machines with extensive arrays, significantly expanding the allowable working area.

On the software side, a continuous polyline generated in CAD was imported into the proprietary software WIREpath version 1.0.7 [11]. This software allows for modifications and conversion of the polyline into a G-code format that the Tajima machine can recognize. The Tajima machine then utilizes this continuous polyline as a tool path for tailoring flax fiber fed from a bobbin. Despite the continuity of the CAD file, instances arise where the length of the tool path exceeds the length of the fiber on the bobbin.

To maintain fiber continuity, segments of fiber are intentionally overlapped onto the substrate. This principle is consistently applied when extending fiber paths beyond the boundary region of a tailoring head. The use of the shared 180 mm wide overlapping region, accessible from both tailoring heads, results in the creation of dual files. These files are designed to deliberately overlap fibers, ensuring a continuous and uninterrupted fiber path. However, it is important to note that a limitation of this technique is that the maximum size of the NFRP sheet is constrained by the size of the substrate mounting frame.

**Figure 5:**
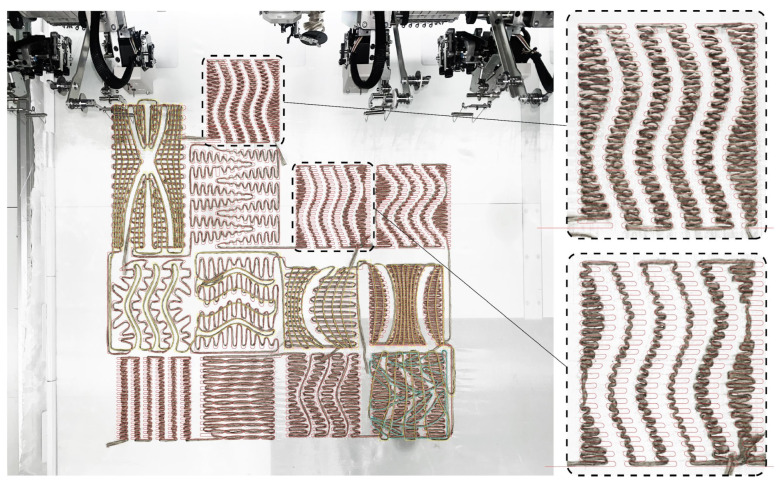
Example of acute angle direction change in embroidered fiber causing physical deviation from digital path.

Initial samples were designed with different patterns to assess the viability of the curved folding system with the selected materials (Figure 5). The fabrication of these samples provided insights into the discrepancies between the physical samples and the digital patterns (generated with McNeel’s Rhinoceros 3D version 7.0). Notably, the most significant variance between the physical and digital paths occurred when the tailored fiber changed direction to create acute angles, as depicted in Figure 5. To address this challenge and to investigate unknown variables and tolerances of the machine, additional stitch points were strategically incorporated into the machine G-code within the proprietary software. This adjustment effectively reduced the margin of error by 3 mm in these specific locations.

### 2.3. Compliant Folding

To shape a structurally rigid form from the two-dimensional output of the fabrication process, curved folding is employed. Additionally, curved corrugation is used as a strategy to enhance the resultant geometry’s rigidity. In curved folding, developable surfaces undergo elastic deformation through the use of curved creases [12]. The design of these creases must be coordinated with the resultant developable surfaces. The relationship between crease areas and the resulting shaped form is explored through digital simulation and subsequent rapid prototyping in Section 2.6 and Section 2.7.

**Figure 6:**
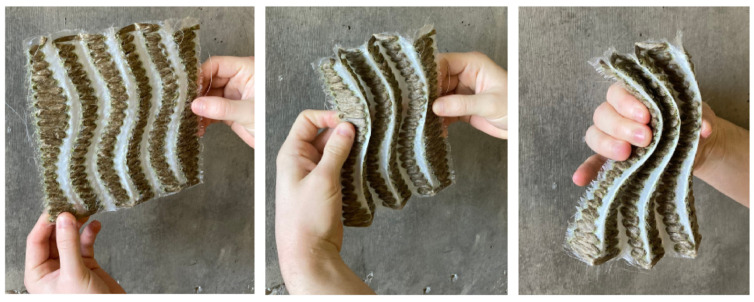
An image sequence of test sample, demonstrating folding along lines created through selective curing.

A technique for compliant folding through the selective curing of the resin matrix was investigated (Figure 6). Flax Fiber was densely tailored to create two-dimensional wavy strips side by side, leaving a gap between each TFP region to serve as the folding zone. This gap marked the regions for the application of a flexible elastomer that serves to prevent the resin from being absorbed by the textile during the infusion process. Building upon the areas of civil and industrials engineering applications of fiber-reinforced rubbers such as tires, dampers, and conveyor belts [13] conventionally known as tailored fiber-reinforced elastomers (TFRE) this process becomes an additional step in the conventional workflow, occurring between the TFP and resin infusion steps.

Five different materials—wax, water-soluble glue, petroleum jelly, thin silicon, and thick silicon—were assessed to create compliant fold zones. After applying these materials to the fold zones, the samples underwent vacuum infusion with resin and were allowed to fully cure. Thin silicon proved to be the most effective in creating a compliant fold zone, due to its ability to repel resin from the substrate. 

The application process involved applying the silicone with a caulking gun to the uncured substrate on both sides of the fabric after tailoring. The silicone was left to dry for approximately one hour, after which the preform was resin infused and left to cure. It was possible to observe that, after curing, the silicone areas remained flexible, while the fibers were completely impregnated and thus rigid. A thin layer of excess resin was observed over the silicone; however, this layer could be easily decoupled from the programmed flexible zone.

Having gained insights into machine tolerances and the innovative process of selectively curing parts of the substrate, the subsequent phase involved prototyping larger samples. The aim was to assess the efficacy of curved corrugated patterns and the capability to accurately fold into a global shape.

### 2.4. Scaled Prototyping

**Figure 7:**
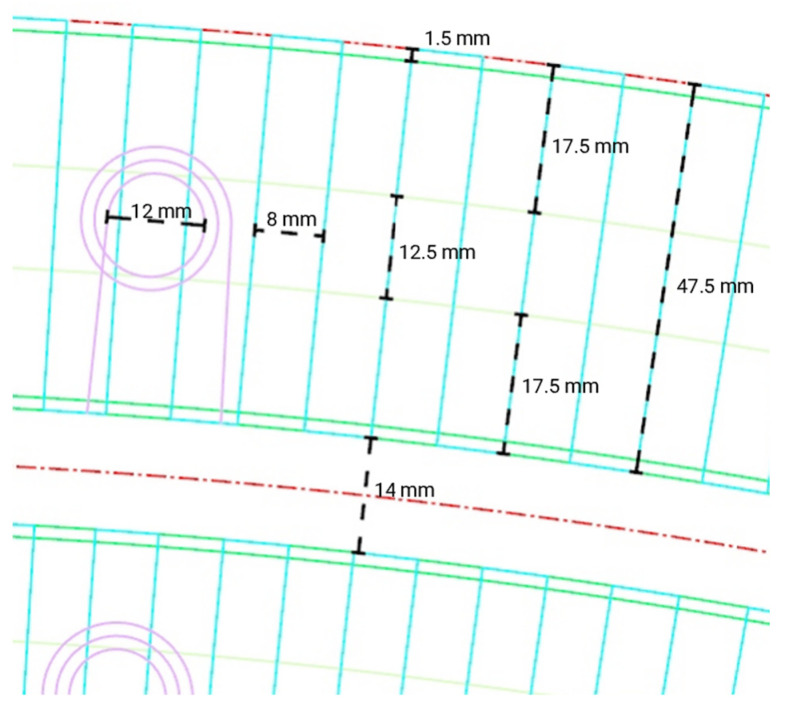
Diagram showing parameters used to control path planning algorithms. The roving paths are colored according to function and layer, note not all elements in the pattern are represented.

The scaled prototypes take geometric inspiration from the curved folding paper projects conducted at the Bauhaus, shaping annuli from flat preforms [14]. These prototypes serve as a proof of concept for the working material system and intentionally reveal potential shortcomings and failures in the methods. To establish a repeatable and efficient workflow, insights from the exploratory NFRP samples (Figure 5) were leveraged to develop a parametric path planning algorithm. This algorithm automatically generates a tight zigzagging infill pattern between inner and outer concentric rings (Figure 7), offset multiple times inwards with gaps, producing a donut preform with a parameterized number of fold zones.

Two iterations of scaled prototypes featuring donut-shaped preforms were created. Both iterations maintained a constant outer diameter of 785 mm, fitting within the maximum working area of a tailoring head. The variations between the two prototypes lay in the number of fold zones and the resulting inner diameter of the donut preforms.

The first annulus featured an inner diameter of 200 mm with five 52 mm wide corrugations. The second annulus gained an additional corrugation and this increased inner diameter increased to 400 mm, making the widths of the corrugations 25 mm wide.

**Figure 8:**
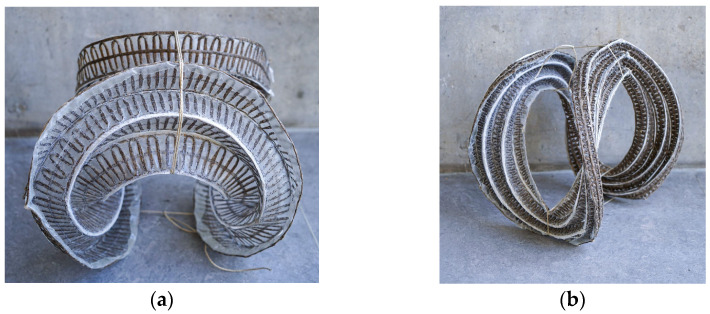
Images of hand bent prototypes furnished to improve methods and design processes. Both prototypes have the same circular outer diameter of 400 mm while testing a variety of other parameters. Prototype (**a**) contains six 5 mm corrugations with 8 mm folding zones. Prototype (**b**) contains five 10 mm corrugations with 5 mm folding zones.

The two annuli were tailored, silicone was applied on the folding zones, and then both samples were subsequently resin infused. Both cured flat preforms were then hand-bent into shape, and dry flax roving was wrapped around the folded corrugations at intervals around the bend piece’s circumference to prevent the preform from reverting to its flat state (Figure 8). These prototypes demonstrated that curved folding patterns could be applied to an NFRP component with silicone-compliant fold zones.

**Figure 9:**
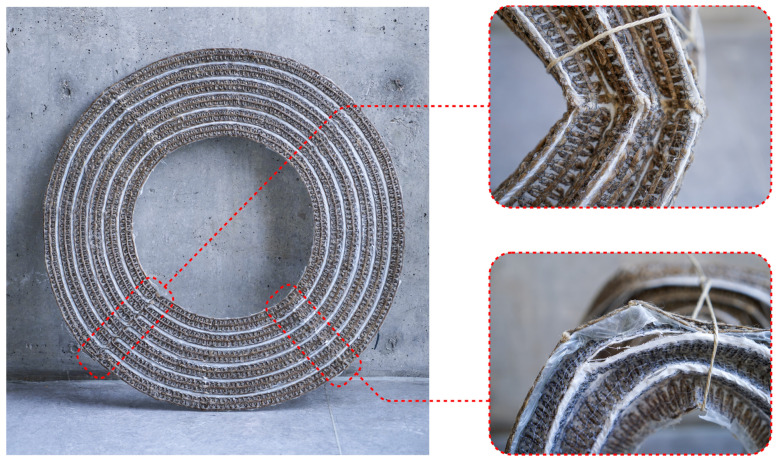
Image of flat packed form of (Figure 8b) prototype with superimposed linework highlighting nodes of identified failure. The callout on the left side shows flaws in the path planning that led to unintended points of folding and fabric failures. The callout on the right side shows an unintended fold line created during the curing process.

The shaped prototypes underwent a load test with the weight of a filled backpack and a piece of plywood, approximately 20 kg combined. While this initial, nonscientific load test did not reveal much about the structural capacity of the bending active system, it provided insights into potential failure modes when upscaling these fabrication methods to larger components (Figure 9). Issues included unintended fold lines caused by the infusion step when folding the uncured tailored piece to fit within vacuum bags during resin infusion. A larger vacuum bag could eliminate the need to fold the uncured piece. Additionally, the twine wrapped around the corrugated form caused unintended breaking of continuous fibers by exerting excessive force on the material in these locations. Integrating holes for drawstrings into the path planning algorithm would offer a better alternative to fastening twine without damaging the NFRP, alleviating additional stress on the already elastically deformed surfaces. The continuous fiber path in the fabrication process led to an uncontrolled opening in the fiber paths and undesirable seams when moving between concentric rings. Removing the fiber segment from the fold zone would provide a continuous channel around the circumference of the piece.

### 2.5. Path Planning

**Figure 10:**
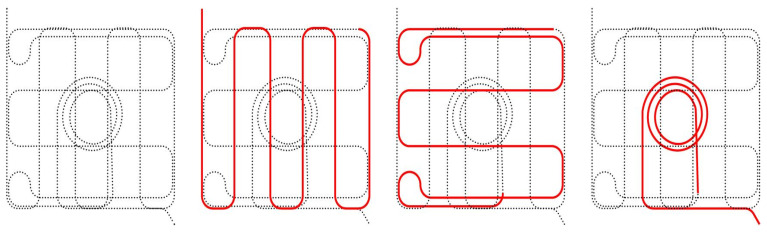
Diagram of isolated parametric paths and their connections. Red lines represent each layer of tailoring onto the substrate.

If the resultant NFRP curve folded geometry was not locked into its shape, the NFRP piece would un-fold back into its flat state. For the initial studies, twine was simply tied around the folded corrugated material; however this led to localized failure. The integration of reinforced eye holes was implemented into the path planning algorithm which would aid in the locking of the curved form with twine. The eye hole syntax is integrated into the single polyline toolpath that generates the entire NFRP sheet (Figure 10). The complete three interconnected layers of continuous fiber paths are meant to program the final folded geometry while also systematically reinforcing areas of high stress and finally placing predetermined penetrations for locking. At specified locations along the zig zagging reinforcement layer, a flat spiral syntax begins by an inside diameter of 6 mm and spirals outward two revolutions before continuing back into the zig zag pattern. In these instances, the center eye hole must be unobstructed from flax fiber. Depending on the design scenario, the spirals’ centers must be adjusted within the zig zag syntax to ensure that there is no overlap. 

A parametric path planning algorithm was developed to rapidly generate variations of the annulus design, considering factors such as dimensionality, the number of corrugations, flax fiber tailored density, and fold zone width. Implemented in Grasshopper 3D, this algorithm ensures that a continuous polyline is dynamically generated by adjusting various input parameters. By automating this process, the algorithm significantly reduces the time required for the manual drafting of linework, offering an efficient solution for generating a G-code for fabrication.

### 2.6. Simulation and Analysis

As the variation between the two prototype iterations (Figure 8) did not provide sufficient insight into their relationship with structural performance, an exploration into a workflow utilizing digital simulation and analysis was undertaken. This approach aimed to predict the behavior of the formed shape, taking into account factors such as physical materiality, load conditions, and geometry.

In the simulation process of curved folding, two techniques were explored. The first involved using a Grasshopper3D plug-in called CRANE [15]. This plug-in generates rigid folds from mountain and valley codified polylines using a built-in solver. To simulate curved folding, the input mountain and valley curves were discretized into line segments.

The second simulation method utilized Kangaroo Physics [16]. In this digital workflow, flat quad meshes served as inputs for the physics simulation. Mesh vertices from the input quad meshes were used to configure the hinging effect, creating mountains and valleys controlled by a parametric rest angle. The same rest angle was applied to both the mountain and valley folds, with the angle made negative for valley folds to invoke a hinge effect in the opposite direction. To prevent form finding from distorting the mesh faces upon folding, an additional goal of edge length was introduced in the simulation. This involved cross bracing each mesh face, setting diagonal lines to remain a constant length.

It was observed that Kangaroo’s resulting mesh geometry provided greater resolution and more consistent results to the physical prototypes over CRANE while also demonstrating superior integration into the overall form-finding design process. Moreover, the same polylines used for creating the quad mesh could be seamlessly integrated into other parametric workflows, such as path planning (2.3 Compliant Folding) and TPU 3D printing for rapid prototyping (2.5 Path Planning).

**Figure 11:**
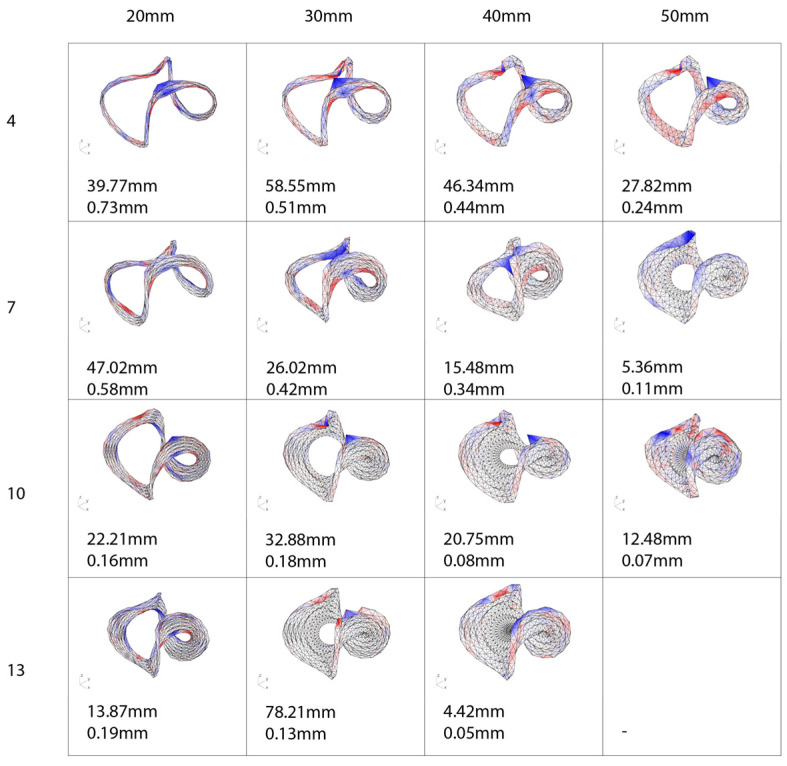
Dataset representing a series of digitally simulated prototypes. The columns indicate the varied corrugation widths, and the rows indicate the number of corrugations for each prototype. Each simulated prototype is represented by a digital graphic showing the deflection heat map and the tabulated maximum and average deflection in mm noted below.

Once the two-dimensional mesh was computationally curve-folded into its three-dimensional form using the physics solver, the simulation was paused, and the resultant mesh was input into Karamba3D [17]. The form was structurally evaluated for maximum deflection relative to the loading scenario. As seen in (Figure 11), the structural defection to a series of study models with varying corrugation width and numbers of corrugations was analyzed. Using the material properties for flax fiber composites available in the literature [18], a material component for Karamba3D is input to allow the mesh to simulate how flax fiber would behave when loaded to 80 kg. From the results in Karamba3D, the input variables fed into Kangaroo Physics were adjusted and the simulation was evaluated again. This process was repeated until we obtained an understanding of the structural performance offered by the number and widths of corrugations within the design of the annulus. The output of the analysis proved that more corrugations or wider corrugations in the design of the folds led to the least amount of deflection when loaded.

### 2.7. Rapid Prototyping via 3D Printing

Although full scale prototyping provided insight into further considerations as noted previously, the methods for rapid prototyping to determine flat to shaped global geometry were explored by augmenting the TFP process through the 3D printing of flexible filament (TPU) onto a stretched fabric clamped over the printer bed.

**Figure 12:**
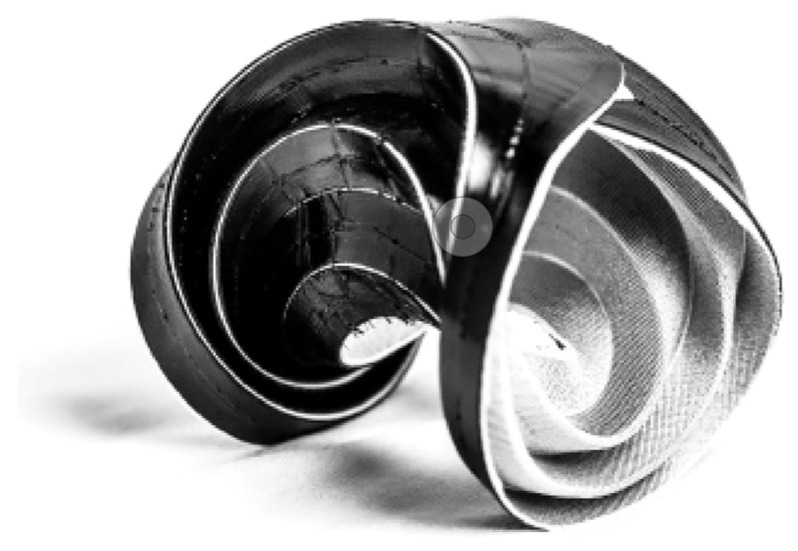
Photograph of final demonstrator made by 3D printing TPU on fabric.

In this fabrication process, the same concentric ring inputs for TFP path-planning generation are used to generate 2 mm tall solid 3D printed extrusions. These extrusions will be printed directly on top of stretched polyester fabric. The TPU filament binds to the fabric, acting as semi-rigid segments in the folding process. Once printed, the excess fabric around the outer and inner diameter of the piece is cut away and the piece is bent into shape (Figure 12). Using this method of prototyping, global geometry can be achieved in about an hour; in contrast, it would take two days to achieve one iteration using the TFP machine.

In conjunction with digital simulation and analysis, TPU rapid prototypes were employed to swiftly iterate through morphological studies of the annulus. This approach also facilitated the exploration of morphologies aimed at securing the stool in place, preventing the geometry from reverting to a flat state. Elongated flaps at opposite ends of the annulus were designed to overlap during the folding process. Once folded, these flaps followed the slight curvature of the geometry, providing additional rigidity. Apart from the material doubling at this location and further reinforcing the load area, the flaps served as the seating surface in the stool demonstration. For full-scale fabrication, similar spiraling holes to those mentioned in Section 2.5 were integrated for drawstrings on these flaps, aligning when folding to secure the geometry in place. However, in the case of 3D-printed rapid prototyping, glue was simply used to lock the overlapped segments.

## 3. Results

Combining the rapid prototyping fabrication method via 3D printing with the digital tools for simulation and analysis, the desired global geometry was achieved. The final geometry was optimized using these methods to fit within the tailoring frame, which could be shaped into a stool with an appropriate seating height and engineered to withstand a specified target load of 80 kg.

**Figure 13:**
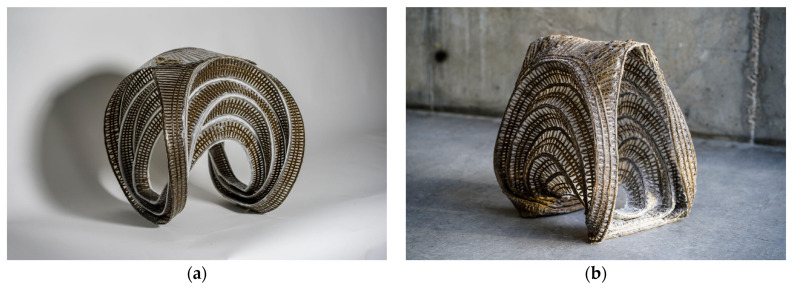
(**a**) Photograph of stool one; (**b**) Photograph of stool two.

**Figure 14:**
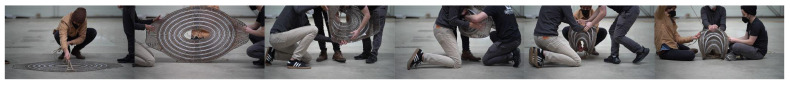
Series of images showing the process of folding a flat preform into its programmed geometry and securing the form with a drawstring. This process can be reversed by simply removing the drawstring.

After establishing the global geometry and validating the structural criteria for the stool designs, two full-scale stools were produced. Maintaining consistency in the stitching settings and fiber materials, variations were introduced solely in the curing and forming processes as part of a benchmarking exercise. Stool one used the methods developed in this research to create a flat cured sheet with silicone hinges that were actively bent into shape (Figure 13a). The deployment of this prototype is depicted in Figure 14. Stool two, on the other hand, lacked silicone hinges and was fully impregnated with resin before being shaped and held in place while the resin was curing (Figure 13b).

**Figure 15:**
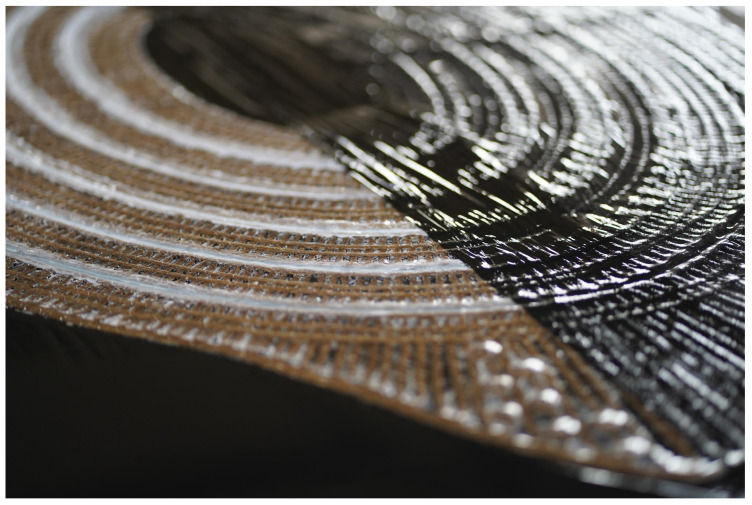
Close up image of a cured flat preform with silicone hinges. During curing, the preform was placed between plastic sheets in a vacuum press table.

Comparing the final results of the two stools from an aesthetic perspective, stool one showcased a consistent and smooth surface finish that aligned with the prerequisite simulations while stool two exhibited a raw surface texture and irregular global form. This is due to the first stool being cured flat inside the vacuum press table (Figure 15). Stool one, utilizing elastic deformation properties, proved that formwork could be entirely eliminated when combined with elastomer pre-curing to create hinges in the NFRP. Inherent to its materiality, the use of NFRP allows for a bending-active product to shape and unshape stool one making DfD and reduced transportation volume possible. In contrast to stool one, stool two was formed using light scaffolding prior to being fully cured. The weight of the fiber and substrate distorted the resultant global geometry.

**Figure 16:**
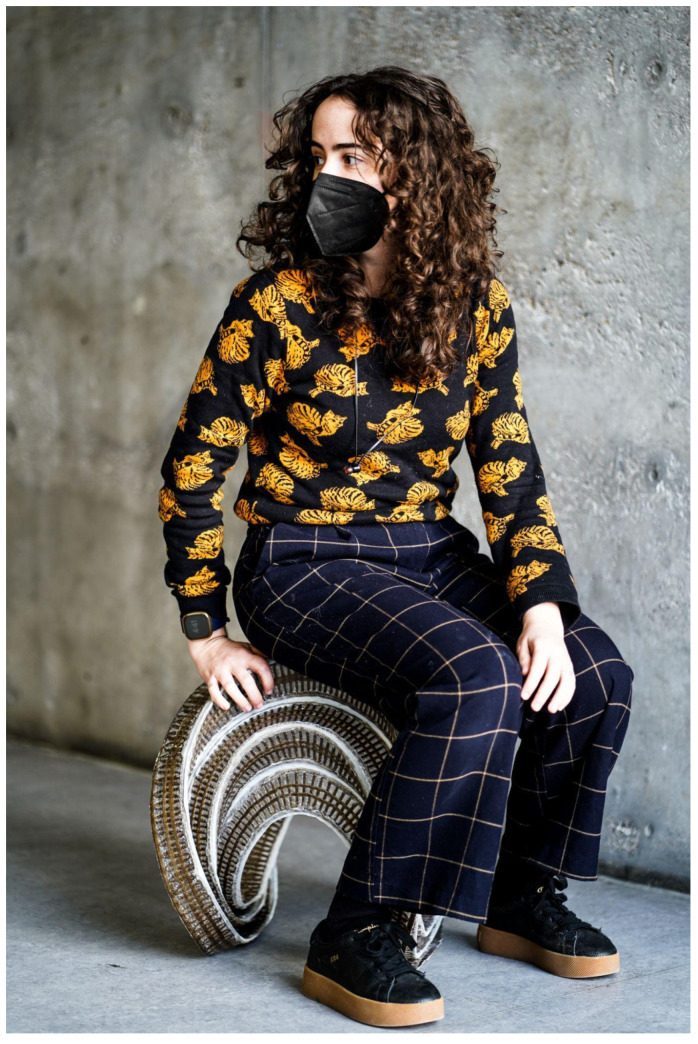
Photograph of stool 1 in use.

Structurally, both stools were able to withstand the weight of a sitting adult (Figure 16). In the actively bent stool, the bending of the NFRP made the form very stiff and exhibited a dampened rebound. However, at the curved folding seams, the silicone and uncured glass fiber composite substrate showed signs of tearing and localized failure. This could be due to the sharp edges of cured resin that being adjacent to the softer, textile-like qualities of the silicone composite folding zones. To resolve this, types of additional fibers, whether they be similar to natural fibers or more typical embroidery threads, could be tailored into folding zones to prevent ripping. In stool two, the NFRP was not actively bent and was far less stiff. When loaded, audible sounds of buckling could be heard.

## 4. Conclusions

**Figure 17:**
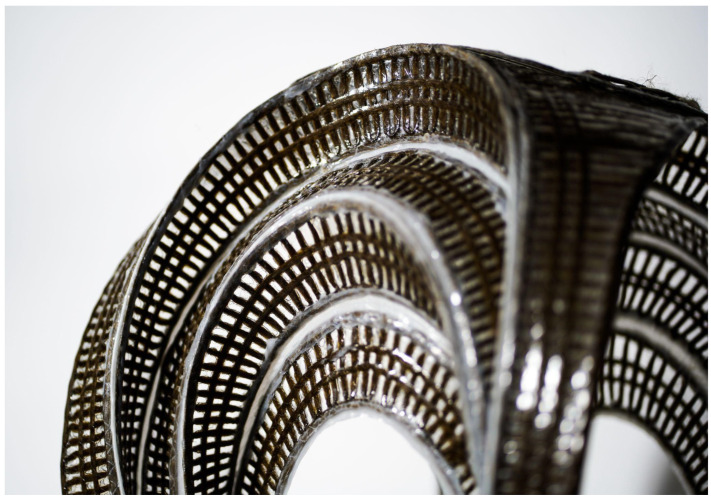
Close up photograph of stool 1.

The final artifact that emerged from this research is proof of concept of curved folding corrugation made with NFRPs in combination with TFRE’s for reversibly deployable architectural-scale structural components (Figure 17). The most significant outcome, apart from the form itself, was the performance of the material in collaboration with geometric programmed deformation. Stool one weighed approximately 1.2 kg and was able to withstand the weight of an 80 kg adult. This is a multiplication factor of nearly 67 which provides evidence of the benefits of using NFRPs for architectural components. This data, however, is highly approximated with the load test being carried out by merely sitting on the stools. Proper load tests should be performed to precisely determine the structural capacity of this curved folding method. 

To bring the methods for curved fiber composite corrugation within this research closer to the built environment, further investigations should consider how larger architectural scale components can be discretized into smaller subcomponents. When bending larger components than those presented in this research, new considerations would need to be implemented to account for the energy and tools needed to form the components. For instance, the stool demonstrators required three workers to fold the flat preform into shape.

A continuation of this research should investigate methods to strengthen the durability of the compliant fold zones. In this research, a glass fiber sheet substrate was infused with silicone to prevent resin impregnation. Although able to successfully create a prototype that was able to be folded and resisted 80 kg of weight when loaded, through wear and tear, slight tears along the fold zones began to emerge. This failure was likely due to the weak structural properties of the glass fiber fabric. Additionally, the abutment of sharp cured glass fiber would dig into and begin to rip into the silicone coating.

Future prototypes should consider a different substrate other than glass fiber to further reduce the carbon footprint and greater enhance the sustainability of the components. Substrates with increased structural properties when combined with elastomers should be tested to improve the resiliency of the components along the fold zones.

FlaxPack introduces a novel method to prefabricate complex geometry with predictable and controlled conditions, efficient flat-packed transportation, and on-site curve-folded deployment and disassembly conducted with the precision offered by robust digital form finding and structural analysis simulation. Two stool designs are compared to demonstrate the feasibility and benefits of flat-packed elastomer hinges combined with NFRP over traditionally formed in place NFRP components. This research highlights the advantages of NFRP for architectural components by showcasing deployable systems with high strength-to-weight ratio, and reducing reliance on formworks, addressing typical drawbacks of traditional methods.

## Data Availability

The original contributions presented in the study are included in the article, further inquiries can be directed to the corresponding author/s.
